# Real-time observation of jumping and spinning nanodroplets

**DOI:** 10.1063/1.5135699

**Published:** 2020-01-14

**Authors:** Pavel K. Olshin, Jonathan M. Voss, Marcel Drabbels, Ulrich J. Lorenz

**Affiliations:** Laboratory of Molecular Nanodynamics, École Polytechnique Fédérale de Lausanne, 1015 Lausanne, Switzerland

## Abstract

The manipulation of liquids at nanoscale dimensions is a central goal of the emergent nanofluidics field. Such endeavors extend to nanodroplets, which are ubiquitous objects involved in many technological applications. Here, we employ time-resolved electron microscopy to elucidate the formation of so-called jumping nanodroplets on a graphene surface. We flash-melt a thin gold nanostructure with a laser pulse and directly observe how the resulting nanodroplet contracts into a sphere and jumps off its substrate, a process that occurs in just a few nanoseconds. Our study provides the first experimental characterization of these morphological dynamics through real-time observation and reveals new aspects of the phenomenon. We observe that friction alters the trajectories of individual droplets. Surprisingly, this leads some droplets to adopt dumbbell-shaped geometries after they jump, suggesting that they spin with considerable angular momentum. Our experiments open up new avenues for studying and controlling the fast morphological dynamics of nanodroplets through their interaction with structured surfaces.

## I. INTRODUCTION

Despite the common occurrence of liquid drops, the fast morphological transformations they undergo in many everyday processes were only revealed once high-speed flash photography became available. Harold E. Edgerton's 1936 “Milk Drop Coronet” is a now iconic example that spectacularly captures the impact of a drop on a liquid surface.^1^ More recently, flash photography has been used to observe the dynamics of highly charged micrometer-sized droplets undergoing Coulomb fission,^2,3^ elucidating a complicated fluid dynamics problem central to the electrospray ionization process.^4–7^ Due to the shorter length and faster time scales involved, such approaches cannot be extended to study nanodroplets—ubiquitous objects whose controlled generation and manipulation is crucial to a number of technologically important endeavors,^8^ such as jet printing with droplets of ever smaller dimensions.^9^ At the nanoscale, fluid dynamics may be significantly altered by specific nanoscale and surface effects,^10–12^ making real-time observations crucial for developing a detailed understanding of these systems.

Here, we study the formation of so-called jumping nanodroplets, which are created when metal nanoprisms are flash-melted with a laser pulse.^13,14^ On a surface that repels the liquid metal, dewetting ensues and the nascent nanodroplet jumps off its substrate, reaching velocities of about 20 m/s. This process is closely related to a broader range of fundamentally important phenomena, including the dewetting of extended liquid films,^15^ the coalescence-induced jumping of water droplets from the hydrophobic surface of self-cleaning lotus leaves,^16^ and the process of droplets impacting a surface.^17^ Recently, jumping droplets have even been used to fabricate large arrays of uniform nanoparticles.^18^ The mechanism of this process has been investigated both by molecular and continuum dynamics simulations.^19–21^ Molecular dynamics simulations have recently also uncovered a related phenomenon in which nanodroplets are propelled from a structured graphene surface.^22^ However, in the absence of real-time observations, our understanding of the dynamics is incomplete.

We can estimate the time scale of the dewetting process using Rayleigh's theory of liquid droplet oscillations.^23^ The progression of the droplet from a flattened to a prolate shape that propels itself off the surface resembles a half-oscillation in the fundamental mode of the droplet with the period τ given by

$$\tau =\sqrt{\frac{\rho r^{3}\pi^{2}}{2\sigma }},$$ (1)

where r is the droplet radius, ρ is the density, and σ is the surface tension of the liquid.^23^ For example, a liquid gold droplet of 270 nm diameter, which is the size of the droplets studied here, has a half-period of about 7 ns.

## II. EXPERIMENTAL SETUP

**FIG. 1.**
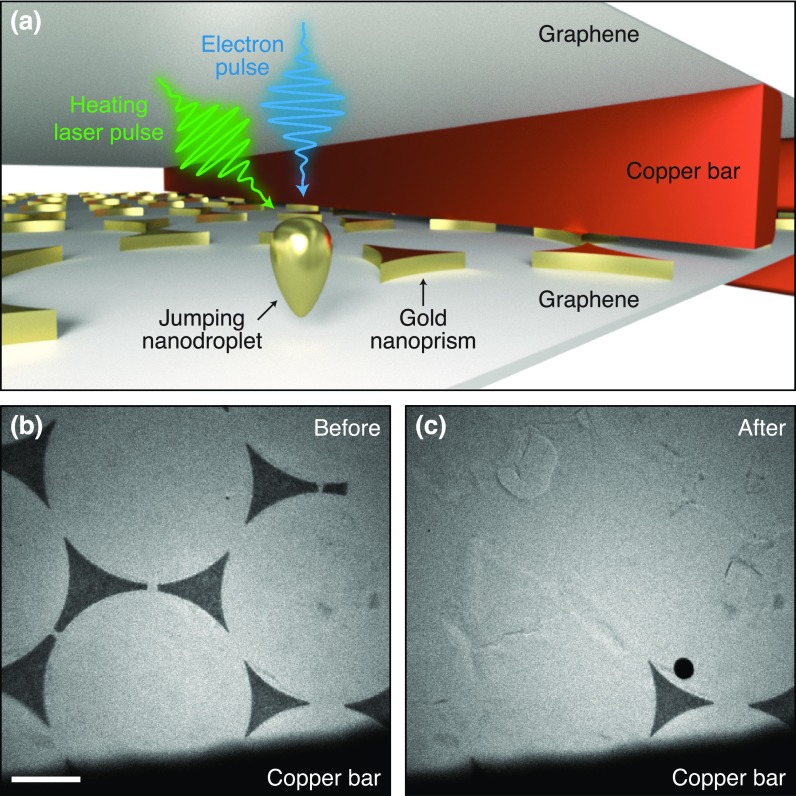
Gold nanoprisms as precursors of jumping nanodroplets. (a) Illustration of the sample geometry. Multilayer graphene decorated with gold nanoprisms is supported by an amorphous carbon/Formvar thin film on a copper mesh. A second copper grid covered with graphene is added on top. (b) Micrograph of nanoprisms before and (c) after flash-melting with a laser pulse. (Scale bar, 1 *μ*m.).

In order to directly observe jumping droplets of such small dimensions and on such short time scales, we employ time-resolved electron microscopy.^24–27^ We perform experiments with a modified JEOL 2200FS transmission electron microscope that we operate with an accelerating voltage of 160 kV. Briefly, a short laser pulse illuminates the sample to initiate the dynamics, which are subsequently imaged with a short electron pulse at a specific time delay. The electron pulse (∼2 × 10^4^ electrons, 1 ns) is generated by illuminating the extractor electrode of the electron gun with a UV laser pulse (266 nm, 1 ns FWHM, 2 *μ*J). This enables us to capture the morphological evolution of individual droplets with single nanosecond time resolution, more than an order of magnitude higher than previously achieved in related experiments on nanoscale fluid dynamics.^26,28,29^ Figure 1(a) shows a sketch of the sample geometry used in our experiments. As precursors for the jumping nanodroplets, we use triangular gold nanoprisms (990 nm side length, 30 nm height). As in previous studies of jumping nanodroplets, the nanoprisms were fabricated with colloidal lithography.^30–32^ The nanoprisms reside on a multilayer graphene film, which is supported by an electron microscopy specimen grid (amorphous carbon/Formvar film on 300 mesh copper). Another sample grid (multilayer graphene on 2000 mesh copper) tops the assembly. It reduces the total fluence incident on the thin film substrate that otherwise tends to rupture, and it prevents droplets from contaminating the instrument. A micrograph of a typical sample is shown in Fig. 1(b). Illumination *in situ* with a short laser pulse (515 nm, 3 ps FWHM, 1 *μ*J, focused to a spot size of 32 *μ*m FWHM) melts the structures and generates jumping nanodroplets. After the laser pulse, most of them disappear from view, and only a single spherical nanoparticle can be observed on the substrate [Fig. 1(c)]. Its diameter (310 nm) is larger than expected for a droplet resulting from a symmetric nanoprism (270 nm), suggesting that the droplet either deformed upon impact and solidified in a flattened shape or it originated from a larger structure not visible in the micrograph in Fig. 1(b). Figure 1(c) shows that some nanoprisms close to the copper bars remain. Since the melting laser pulse strikes the sample at a small angle with respect to the electron optical axis, these structures are likely partially shadowed by the copper bars. We also occasionally find that droplets resolidify on the uppermost graphene layer upon impact (Fig. S1).

## III. RESULTS AND DISCUSSION

**FIG. 2.**
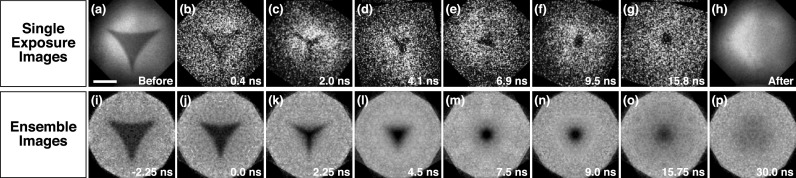
Snapshots of jumping nanodroplets and ensemble images. (a) A nanoprism is melted with a short laser pulse, and the (e) jumping nanodroplet is imaged at 6.9 ns with a single electron pulse. (h) After the jump, it leaves behind the bare substrate. (b)–(g) Representative snapshots of other nanodroplets at different stages of the dewetting process. (i)–(p) Ensemble averaged images of the dynamics (scale bar, 500 nm).

We elucidate the fast fluid dynamics following the melting laser pulse by recording snapshots with individual, short electron pulses. Flash-melting the nanoprism in Fig. 2(a) creates a jumping nanodroplet that disappears from view [Fig. 2(h)]. A snapshot recorded at 6.9 ns reveals the transient structure of the collapsing droplet, which has contracted into a compact mass but has not yet assumed a spherical geometry [Fig. 2(e)]. By repeating the experiment on other identical nanoprisms and taking snapshots at different time delays, we capture the entire evolution of the droplet morphology [Figs. 2(b)–2(g)].

It is informative to analyze the dynamics both in terms of the average behavior of the ensemble and the variations of individual trajectories. We obtain a movie of the ensemble dynamics by averaging over 500 individual exposures within 0.75 ns time-bins [Figs. 2(i)–2(p), Fig. S2, and Movie S1]. In this manner, we overcome the low signal-to-noise ratio afforded by an individual exposure, which results from the low number of electrons available in a single pulse. Averaging requires the objects to be aligned to each other, which we achieve with the following procedure. Before melting with a laser pulse, each nanoprism is imaged with an accumulated dose of 1000 electron pulses. The resulting high signal-to-noise micrograph is then aligned to a reference using phase correlation-based image registration.^33^ The same rigid transformation is then applied to the time-resolved image of the jumping droplet that was subsequently recorded with a single electron pulse. In order to account for intensity variations of the electron beam, the time-resolved image is normalized by another micrograph (1000 electron pulses) of the bare specimen support that is left behind after the droplet has jumped and disappeared from view. Finally, the time-resolved micrographs are sorted into 0.75 ns time-bins, symmetrized assuming C_3v_ symmetry, and averaged to yield the ensemble images of Figs. 2(i)–2(p).

These ensemble images reveal that after flash-melting, the triangular structure immediately begins to collapse onto its center of mass [Fig. 2(j)]. Within 2 ns, the prism sides have retracted inward, so that the droplet transiently adopts a star-shaped configuration [Fig. 2(k)]. As the vertices continue to be pulled inward, the droplet develops a more compact, triangular outline [Fig. 2(l)], which approaches a circular shape around 7 ns [Figs. 2(m) and 2(n)]. At longer times, the droplet outline increasingly blurs [Figs. 2(o) and 2(p)]. The observed morphological evolution qualitatively agrees with both molecular and continuum dynamics simulations.^19–21^ For gold nanoprisms of 47 nm thickness and 405 nm side length on a silicon dioxide substrate, the collapsing droplet was predicted to maintain a triangular outline, whereas thinner nanoprisms (24 nm) adopted the transient star-shaped configuration we observe in our experiments.^21^

**FIG. 3.**
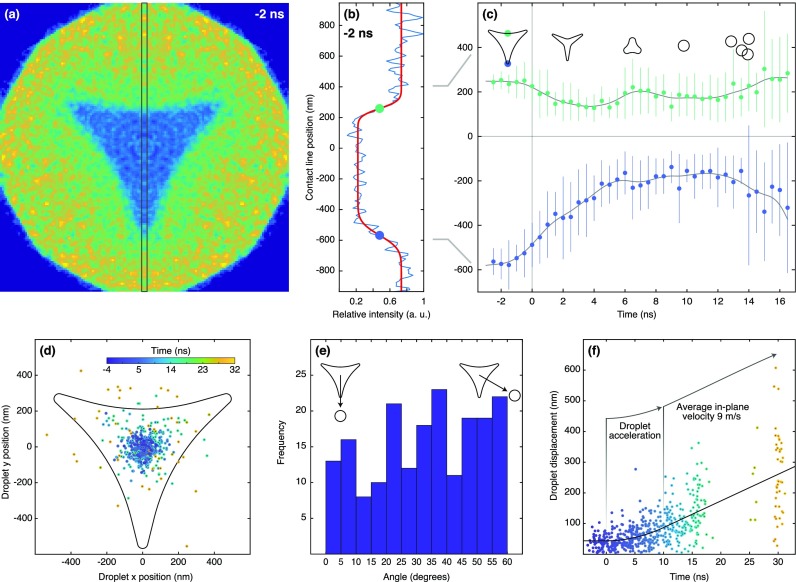
Analysis of the jumping nanodroplet dynamics. (a) Ensemble image of the nanoprism 2 ns before flash-melting. (b) Intensity profile (blue line) of the area marked in (a). The center positions of the boundaries (dots) are extracted from a fit (red line). (c) Center positions of the droplet boundaries as a function of time (dots). Vertical lines indicate the standard deviation of the average droplet boundary position (supplementary material Note 1). The gray lines serve as a guide for the eye. (d) Positions of the center of mass of the jumping droplets at different time delays (encoded by the color scale, inset). (e) Angular distribution of the jumping droplets in the time interval of 12–32 ns. (f) The displacements of the center of mass of droplets as a function of time (circles) is fit with a simple model (black line).

We analyze the dewetting dynamics as illustrated in Fig. 3(a). For each ensemble image, we calculate an intensity profile within the marked area and fit it with the product of two error functions [Fig. 3(b), see supplementary material note 1 for details]. This fit yields the average position of the droplet boundaries (dots), which are plotted in Fig. 3(c) as a function of time. The gray lines serve as a guide for the eye and represent splines of the data points. We find that the vertices, which exhibit the smallest radius of curvature and therefore the highest Laplace pressure, retract the fastest (bottom curve), reaching a speed of ∼80 m/s within the one nanosecond resolution of the experiment. On the same time scale, the prism sides (top curve) reach a maximum speed of ∼40 m/s. After 4 ns, the sides reverse direction and expand again until 6.5 ns. Individual snapshots at these time delays reveal inverted triangular outlines [Fig. 2(e)]. This inversion has been predicted to occur after the vertices have collapsed onto the center of mass^20,21^ and is associated with a threefold symmetric in-plane oscillation of the droplet, which then dampens out in less than one oscillation period. At long times (>10 ns), the droplet boundaries appear to move apart again. This is in fact due to a blurring of the ensemble images [Figs. 2(o) and 2(p)], which occurs as the droplets jump in different directions.

The fact that the droplets do not jump straight up but instead have finite in-plane velocities is also evident in Fig. 3(d), which displays the position of individual droplet centers of mass at different time delays (encoded in color). After detaching from the graphene surface, the droplets increasingly spread out without any obvious angular preference [Fig. 3(e)]. Figure 3(f) shows the absolute displacement of the droplets from the center of the nanoprism precursor as a function of time. At negative times, the scatter of the data reflects the accuracy with which we can determine droplet centers from single-exposure images. After flash-melting, the scatter increases rapidly as the droplets contract and finally jump. Assuming a constant average acceleration, a, the in-plane velocity increases linearly until the jump, so that we can fit the droplet displacement Δr(t) with the piecewise function

$$\Delta r\left(t\right)=\left\{\begin{array}{cc} r_{0} & \text{for}\;t\leq 0\\ r_{0}+\frac{1}{2}at^{2} & \text{for}\;t>0\;\text{and}\;t\leq t_{\mathrm{jump}}\;\\ r_{0}+\frac{1}{2}at_{\mathrm{jump}}^{2}+at_{\mathrm{jump}}\left(t-t_{\mathrm{jump}}\right) & \text{for}\;t>t_{\mathrm{jump}}, \end{array}\right.$$ (2)

where $r_{0}$, a, and $t_{\mathrm{jump}}$ are fit parameters [solid line in Fig. 3(f)]. We thus estimate that the droplets detach at $t_{\mathrm{jump}}=$ 10 ns. For comparison, theory predicts droplets to detach at 7 ns (5 ns) for smaller nanoprisms of 405 nm side length and 47 nm (24 nm) thickness.^21^ From the fit, we obtain an average in-plane velocity of the detached droplets of $at_{\mathrm{jump}}=$ 9 m/s, with some droplets reaching more than 25 m/s. Such large in-plane velocities point to substantial asymmetry of the dewetting process. In contrast, a fully symmetric contraction should create a droplet that jumps straight up.^19–21^ Evidently, while the average behavior of the ensemble agrees with theoretical predictions, the trajectories of individual droplets exhibit a certain degree of variation.

**FIG. 4.**
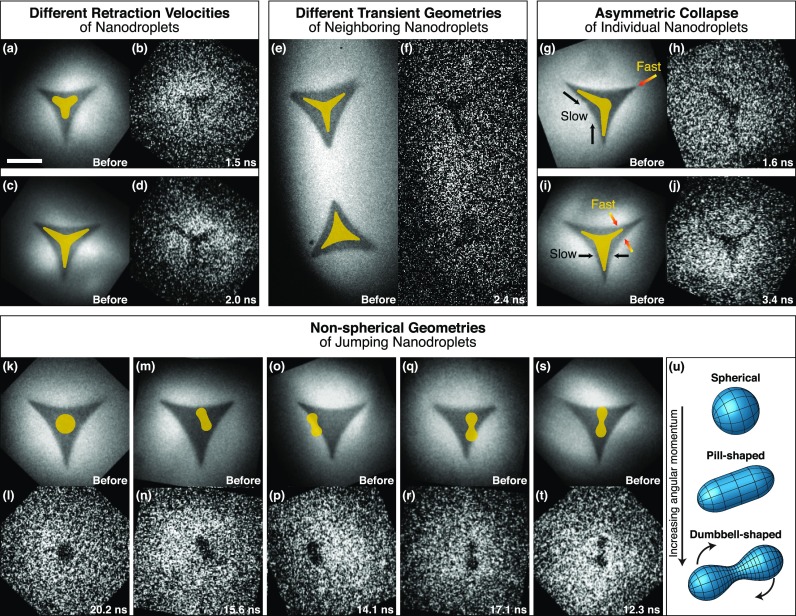
Heterogeneity of the nanoscale fluid dynamics and non-spherical geometries of free droplets. (a)–(d) Snapshots reveal different contraction velocities for different nanoprisms. (e) and (f) Simultaneously flash-melted nanoprisms adopt different transient geometries. (g)–(j) Individual nanoprisms reveal asymmetric transient configurations. (k)–(t) Snapshots of droplets after they have jumped reveal elongated as well as symmetric two-lobed geometries. Outlines of the transient configurations are superimposed on the images recorded before flash-melting (scale bar, 500 nm). (u) Experimentally determined geometries of spinning macroscopic droplets, interpolated from photographs in Ref. 39.

Single-exposure images of jumping nanodroplets indeed reveal such variations in the trajectories of individual droplets (Fig. 4). The nanoprisms in Figs. 4(a) and 4(c) clearly contract with different speeds, as evidenced by snapshots recorded at similar time delays [Figs. 4(b) and 4(d)]. For reference, outlines of the transient structures are superimposed on the images of the nanoprism precursors in Figs. 4(a) and 4(c). Even though the two neighboring nanoprisms in Fig. 4(e) are simultaneously flash-melted, a micrograph recorded at 2.4 ns reveals that they adopt different transient configurations, one of them more star-shaped, the other rather triangular [Fig. 4(f)]. Strikingly, even the collapse of a single nanodroplet can be highly asymmetric, as observed for the nanoprisms of Figs. 4(g) and 4(i) in the snapshots of Figs. 4(h) and 4(j), respectively.

We can exclude that the linear polarization of the heating laser induces the asymmetric nanodroplet geometries we observe. If we take the laser polarization into account when we align and average the single exposure images, we do not observe any significant asymmetry within experimental accuracy (Fig. S3). This suggests that any temperature differences induced by the linear laser polarization do not significantly alter the dynamics. Moreover, molecular dynamics simulations predict that even over a large temperature range of 700 K, dewetting time scales change only nominally,^19^ so that small variations in the droplet temperature should not have a significant effect. We can also exclude that the melting process interferes with the fluid dynamics. At the high temperatures that the nanoprisms initially reach after the laser pulse (heat transfer simulations in Fig. S4), heterogeneous melting of gold is known to occur in approximately 10 ps (Ref. 34), which is fast on the time scale of the dynamics studied here.

Instead, our observations point to friction as the origin of the observed heterogeneity. On a nonideal surface, the moving contact line experiences friction as it dissipates energy through pinning and depinning on defects.^35,36^ Such defects can result from surface roughness as it is present on the multilayer graphene surface used here. The thickness of the graphene substrate varies between six and eight layers on micrometer length scales. Therefore, many nanoprisms span the boundary of a graphene flake, so that the contact line has to move over a microscopic step as the droplet contracts. Defects can also arise from chemical heterogeneity of the substrate, which locally alters the chemical properties of the surface and thus the contact angle of the liquid.^35,36^ A likely source of chemical defects is the colloidal lithography process that we have used to prepare the nanoprisms, following the methodology of previous studies of jumping nanodroplets.^13,14^ This process likely creates microscopic contaminants that locally alter the surface properties. We note that the angular distribution of the jumping droplets does not show any significant anisotropy [Fig. 3(e)], suggesting that the kinetic energy dissipated by each of the three retracting vertices varies independently.

Theoretical studies of jumping nanodroplets have so far only considered idealized surfaces and have therefore neglected the role of friction. In this respect, it is not surprising that on a realistic surface, friction will alter the trajectories of individual droplets and render the dynamics heterogeneous. However, we also observe that the interaction with surface defects gives rise to novel phenomena. Surprisingly, some of the droplets adopt non-spherical geometries in free space. After the droplets have detached from the surface (>10 ns), most appear round as expected [Figs. 4(k) and 4(l)]. However, a small fraction (approximately 5%) clearly features either elongated or very distinct symmetric two-lobed shapes [Figs. 4(m)–4(t)]. It is particularly striking that twofold symmetric droplets are created from nanoprisms with threefold symmetry.

The non-spherical droplets in Figs. 4(m)–4(t) were captured at such late times that they must have already detached from the substrate. Interactions with the surface are therefore absent and cannot explain their geometry. In fact, we do not observe the symmetric two-lobed shapes at earlier time delays when the droplet is still in contact with the surface. Instead, this geometry is unique to droplets in free space. It is also unlikely that the non-spherical droplet shapes arise from large-amplitude oscillations.^37^ Although simulations predict that the droplets oscillate as they jump off the surface, these oscillations occur in the vertical direction and are of much smaller amplitude. They also rapidly dampen out as the droplet relaxes into a spherical geometry.^19–21^ This is not consistent with our observation of symmetric two-lobed geometries that persist at much later times.

Rather, it appears that the two-lobed shapes we observe correspond to the equilibrium geometries of spinning droplets.^38,39^ When angular momentum is imparted on a spherical droplet, it first deforms into an oblate equilibrium geometry and above a critical angular momentum, adopts a pill-like shape with twofold symmetry [Fig. 4(u)]. At even higher angular momenta, the droplet evolves further into a dumbbell shape, as illustrated in Fig. 4(u). We note that these pill and dumbbell shapes^38,39^ bear close resemblance to the geometries observed in our experiments. Very recently, such geometries have been inferred for liquid helium nanodroplets from their diffraction patterns.^40–43^ In our experiments, the angular momentum imparted on the droplet likely has its origin in surface imperfections. On an ideal surface, the C_3v_ symmetry of the system dictates that the droplet cannot acquire angular momentum. However, the interaction of the liquid with surface defects breaks the symmetry of the system. Evidently, the resulting asymmetry of the dewetting process can generate sufficiently large angular momentum, so that dumbbell-shaped spinning droplets are formed.

For such dumbbell-shaped droplets, a unique relationship exists between the length R of the longest axis and the angular velocity Ω (Refs. 38 and 39). This allows us to estimate the rotational period of the droplets if we assume that the longest axis is perpendicular to the electron beam. For the droplets in Figs. 4(p), 4(r), and 4(t), where this appears to be the case in good approximation, we measure R = 180, 220, and 230 nm, respectively. We find that the relative elongations of the droplets $R^{*}=R/r$ do indeed fall within the range for which dumbbell shapes represent the most stable droplet geometry, $R^{*}\approx 1.1-\;2.1$. Through comparison with the numerical results of Ref. 38, we obtain rotational periods of approximately 26, 30, and 31 ns, respectively. We note that the single nanosecond resolution of our experiment is therefore sufficient to resolve the characteristic shape of the spinning droplets.

## IV. CONCLUSION

Our experimental approach opens up new avenues for studying the fast morphological dynamics of nanodroplets through direct observation with time-resolved electron microscopy. To overcome the low number of electrons available in an electron pulse of sufficiently short duration, we average multiple snapshots of identical objects and obtain high signal-to-noise images of the dynamics. This approach should allow the investigation of the faster dynamics of even smaller droplets, for which nanoscale effects will become increasingly pronounced. Our real-time observations add details to the mechanism of the jumping droplets, highlighting the role that surface imperfections play in steering the dynamics. Since by its very nature friction is a nanoscale phenomenon,^44^ such studies of friction on its inherent length scale promise nanoscopic insights that are inaccessible to macroscopic experiments.^35,36^ An intriguing prospect would be to directly observe the interaction of the moving nanodroplets with a single, well-defined nanoscale defect that is lithographically written onto the surface. For example, molecular dynamics simulations have recently shown that water nanodroplets can be accelerated to high velocities when placed on a wedge-shaped defect on a graphene surface.^22^ Here, we present evidence that surface defects can be employed to spin nanodroplets to high angular momenta, suggesting new ways of manipulating these nanoscale systems. For macroscopic jumping droplets, the use of patterned surfaces to induce angular momentum has only recently been demonstrated.^45^

## SUPPLEMENTARY MATERIAL

See the supplementary material for TEM images of droplets resolidified on the uppermost graphene layer, ensemble images of the dynamics, full caption for the movie, note on fitting nanodroplet boundaries, analysis of dynamics for different laser polarizations, heat transfer simulation of droplet temperature, and movie of ensemble dynamics.

## AUTHOR CONTRIBUTION

P. K. Olshin and J. M. Voss contributed equally to this work.
